# Comparative Genome Analysis of the Photosynthetic Betaproteobacteria of the Genus *Rhodocyclus*: Heterogeneity within Strains Assigned to *Rhodocyclus tenuis* and Description of *Rhodocyclus gracilis* sp. nov. as a New Species

**DOI:** 10.3390/microorganisms10030649

**Published:** 2022-03-18

**Authors:** John A. Kyndt, Fabiola A. Aviles, Johannes F. Imhoff, Sven Künzel, Sven C. Neulinger, Terrance E. Meyer

**Affiliations:** 1 College of Science and Technology, Bellevue University, Bellevue, NE 68005, USA; avilesacosta7766@gmail.com; 2 GEOMAR Helmholtz Centre for Ocean Research Kiel, RD3 Marine Symbioses, Düsternbrooker Weg 20, 24105 Kiel, Germany; jimhoff@geomar.de; 3 Max Planck Institute for Evolutionary Biology, 24306 Plön, Germany; kuenzel@evolbio.mpg.de; 4 omics2view.consulting GbR, 24118 Kiel, Germany; sven.neulinger@web.de; 5 Department of Biochemistry, University of Arizona, Tucson, AZ 85721, USA; temeyer@email.arizona.edu

**Keywords:** *Rhodocyclus*, *purpureus*, *tenuis*, *gracilis*, HiPIP, cobalamin, whole genome sequencing, taxonomy

## Abstract

The genome sequences for *Rhodocyclus purpureus* DSM 168^T^ and four strains assigned to *Rhodocyclus tenuis* (DSM 110, DSM 111, DSM 112, and IM 230) have been determined. One of the strains studied (IM 230) has an average nucleotide identity (ANI) of 97% to the recently reported genome of the type strain DSM 109 of *Rcy. tenuis* and is regarded as virtually identical at the species level. The ANI of 80% for three other strains (DSM 110, DSM 111, DSM 112) to the type strain of *Rcy. tenuis* points to a differentiation of these at the species level. *Rcy. purpureus* is equidistant from *Rcy. tenuis* and the new species, based on both ANI (78–80%) and complete proteome comparisons (70% AAI). Strains DSM 110, DSM 111, and DSM 112 are very closely related to each other based on ANI, whole genome, and proteome comparisons but clearly distinct from the *Rcy. tenuis* type strain DSM 109. In addition to the whole genome differentiation, these three strains also contain unique genetic differences in cytochrome genes and contain genes for an anaerobic cobalamin synthesis pathway that is lacking from both *Rcy. tenuis* and *Rcy. purpureus*. Based on genomic and genetic differences, these three strains should be considered to represent a new species, which is distinctly different from both *Rcy. purpureus* and *Rcy. tenuis*, for which the new name *Rhodocyclus gracilis* sp. nov. is proposed.

## 1. Introduction

The anaerobic anoxygenic photosynthetic betaproteobacteria are represented by a small group of bacteria currently classified in the *Burkholderiales* (the genera *Rhodoferax* and *Rubrivivax*) and *Rhodocyclales* (the genus *Rhodocyclus*) orders. Genome sequences of several of the *Burkholderiales* species have been previously reported, including strains of *Rubrivivax gelatinosus* [1,2], *Rubrivivax benzoatilyticus* [3], *Rhodoferax fermentans* [4], *Rhodoferax antarcticus* [5], and *Rhodoferax jenense* [6]. There also is a genome sequence for the aerobic anoxygenic photosynthetic bacterium *Roseateles depolymerans* [7], which is related to *Rubrivivax gelatinosus*.

Though the genome sequence of the type strain of *Rhodocyclus tenuis* DSM 109^T^ has been reported recently [8], the diversity of this group of bacteria has not been studied in detail. The first and type species of the genus *Rhodocyclus, Rhodocyclus purpureus,* was discovered by Norbert Pfennig [9]. A second species of this genus was isolated and described based on a single strain as *Rhodospirillum tenue* [10] and later reclassified as *Rhodocyclus tenuis* [11]. Several strains that were assigned to *Rhodocyclus tenuis* have been isolated from various German freshwater lakes (Lake Pluss-See near Plön, a forest ditch near Grünenplan, Garrensee near Ratzeburg, Eutiner See, Nonnenmattweiher near Neuenweg Black Forest, Edebergsee near Plön) and peat bogs (Black Forest) by Hanno Biebl and Norbert Pfennig [12]. A single isolate obtained from a pond (“keyhole pond”) in the Botanical Garden in Bonn was obtained by one of us (JFI). Parts of these strains have been studied previously regarding to their physiological properties [12], carotenoids [13], lipopolysaccharide structures [14], sulfate assimilation [15], and lipid and fatty acid composition [16]. Although these studies revealed the heterogeneity of the group, no systematic studies have been performed.

Here, we report on the genome analysis of *Rhodocyclus purpureus* DSM 168^T^ and *Rhodocyclus tenuis* DSM 109^T^ and four strains previously assigned to *Rhodocyclus tenuis* and provide evidence for the existence of a new species of *Rhodocyclus,* represented by three of the strains studied that have previously been shown to be different from the type strain of *Rcy. tenuis* [12,13,14].

## 2. Materials and Methods

### 2.1. Origin of the Strains of Rhodocyclus Tenuis

The type strain DSM 109^T^ (Pfennig 2761) was isolated from a pond near Grünenplan (district Holzminden, Germany); strains DSM 110 (Pfennig 3760) and DSM 111 (Pfennig 3761) from Nonnenmattweiher pond near Neuenweg in the rural district Lörrach in the Black Forest (Germany); and strain DSM 112 (Pfennig 3661) from a kolk in the Hinterzartener Moor in the Black Forest, Germany. Strain IM 230 (Imhoff 230) was isolated by JF Imhoff from a small pond called “Schlüssellochteich” (keyhole pond) in the Botanical Garden of Bonn University.

### 2.2. Genome Sequencing

Genomic DNA for *Rhodocyclus tenuis* DSM 110, DSM 111, and DSM 112 was obtained from DSMZ. Cells of *Rcy. purpureus* DSM 168^T^ and *Rcy. tenuis* IM 230 were grown under the recommended conditions [17] and DNA extracted from well-grown cultures according to Imhoff et al. [18]. The quantity and purity of DNA were determined using Qubit and Nanodrop instruments and showed 260/280 ratios between 1.80 and 1.90. The DNA libraries were prepared with the Nextera^®^ XT DNA Sample Preparation kit from Illumina (San Diego, CA, USA) following the manufacturer’s protocol.

*Rhodocylus* sp. strains DSM 110, DSM 111, and DSM 112 genomes were sequenced using 500 µL of a 1.8 pM library with an Illumina MiniSeq instrument, using paired-end sequencing (2 × 150 bp). Quality control of the reads was performed using FASTQC within BaseSpace (Illumina, San Diego; version 1.0.0), using a k-mer size of 5 and contamination filtering. The data for each were assembled de novo using SPAdes (version 3.10.0; [19,20]) for DSM 110 and DSM 111 or Unicycler within PATRIC [21,22] for DSM 112. Default k-mer lengths were used for both programs. The genome sequences were annotated using RAST (Rapid Annotations using Subsystem Technology; version 2.0; [23]). An EvalG genome quality analysis, using the checkM algorithm [24], ran during PATRIC annotation and showed an estimated 100% completeness and 0% contamination for each of these genomes.

*Rcy. purpureus* and *Rcy. tenuis* IM 230 genomes were sequenced on an Illumina MiSeq using the MiSeq^®^ Reagent Kit v3 600 cycles sequencing chemistry (Illumina, San Diego, CA, USA), with a cluster density of approximately 1200 K/mm2. Trimmomatic v0.36 [25] was used for read quality filtering. Illumina Nextera XT adapters were removed from the reads. Quality trimming was conducted with a 5-base pair (bp) sliding window, trimming the reads with an average Phred quality score below 30. Read lengths >21 bp after quality trimming were retained. Only single reads (i.e., reads with their mate deleted) were included into downstream analysis. Reads were further checked for ambiguous base calls, as well as for low complexity, using the DUST algorithm [26]. They were filtered accordingly with an in-house R script in Microsoft R Open v3.3.2 (R Core Team 2016). SPAdes v3.10.0 was used for the pre-assembly of the filtered reads [19,20], using default k-mer lengths. Scaffolds ≥ 500 bp of this pre-assembly were subjected to extension and second-round scaffolding with SSPACE standard v3.0 [27]. Scaffolds ≥ 2500 bp were assigned to genome bins by MetaBAT v0.32.4 [28], to ensure draft-genome purity of *Rcy. purpureus* DSM 168^T^. From the two resulting genome bins (3.595 and 1.048 Mbp, respectively), the larger one with a G + C content of 66 mol% was selected as the draft genome of *Rcy. purpureus*. Base coverage was determined with BBMap v36.81 (https://sourceforge.net/projects/bbmap (accessed on 29 May 2017)) [29] for filtered reads unambiguously mapped to the scaffolds of the draft genome. Estimated fold-coverage was calculated as the median base coverage over all scaffold positions.

### 2.3. Whole Genome Comparison

Average percentage nucleotide identity (ANIb) between the whole genomes was calculated using JSpecies [30]. A whole genome-based phylogenetic tree was generated using the CodonTree method within PATRIC [22], which used PGFams (global (cross-genus) protein families) as homology groups. At total of 445 PGFams were found among these selected genomes using the CodonTree analysis, and the aligned proteins and coding DNA from single-copy genes were used for RAxML analysis [31,32]. iTOL was used for tree visualization [33]. A proteome comparison was performed using protein sequence-based genome comparison using bidirectional BLASTP within PATRIC [22]. Average amino acid identities (AAI) values were calculated from the proteome comparison within PATRIC [22], using only bi-directional hits with *Rcy. tenuis* DSM109^T^ as the reference strain. Digital DNA–DNA Hybridization (dDDH) data were obtained using the Type (Strain) Genome Server (TYGS) web server (https://tygs.dsmz.de (accessed on 3 April 2021)) [34]. The program used the distance formula d4 to calculate a similarity based on sequence identity.

For synteny analysis, global PATRIC PGFam families were used to generate comparative genome regions to determine a set of genes that match a focus gene [22]. All *Rhodocyclus* genomes were used in the search and were compared to the DSM 110 genome. The gene set is compared to the focus gene using BLAST and sorted by BLAST scores within PATRIC [22]. The *cbi*X (long cobaltochelatase) gene was used as a focus gene to analyze synteny of the cobalamin synthesis gene cluster.

The multiple sequence alignments for the 16S rRNA, HiPIP, and RuBisCo comparisons were performed using Clustal Omega [35]. All of the 16S rRNA sequences were genome derived. The phylogenetic tree was calculated by the neighbor-joining (NJ) method [36] within JALVIEW [37] and a Newick file was generated. iTOL was used to draw the phylogenetic trees expressed in the Newick phylogenetic tree format [33].

## 3. Results and Discussion

### 3.1. Whole Genome Analysis

The genomic features of six strains of the genus *Rhodocyclus,* including five new genome sequences and the previously sequenced *Rcy. tenuis* DSM 109^T^ genome, were compared (summary in Table 1). Based on the genome size and the G + C content, three groups can be recognized: *Rcy. tenuis* DSM 109^T^ and IM 230 represent group 1 and have an identical G + C content (64.7 mol%) and slightly larger genome size compared to those of group 2, with strains DSM 110, DSM 111, and DSM 112. *Rcy. purpureus* DSM 168^T^ is the only representative of group 3 and has a similar genome size compared to the group 1 strains but a higher G + C content (66.1 mol%).

The Average Nucleotide Identity (ANI) comparison revealed a 97.1% ANI of strains within group 1 and values of 98.8% and higher of strains within group 2 (Table 2). However, the comparison between strains from the two groups shows ANI values below 80%. Applying the arbitrary cutoff value for species differentiation of 95% [30], the two groups should clearly represent different species. *Rcy. purpureus* has ANI values of 80% or less with all of the other strains and is rightfully recognized as a distinct species. For comparison, *Accumulibacter phosphatis* was included (Table 2) as a species from a closely related genus [38], which showed ANI values of 72–73% with all of the *Rhodocyclus* strains.

**Figure 1:**
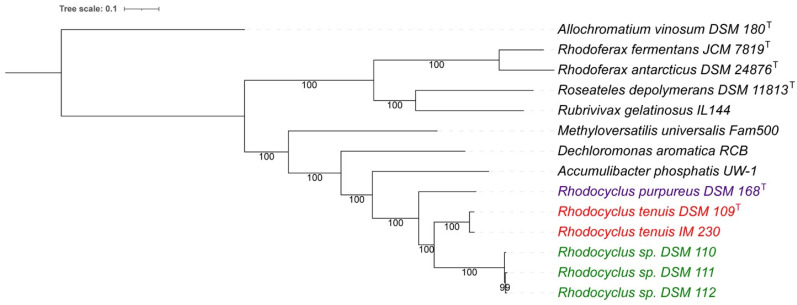
Whole-genome-based phylogenetic tree of all known *Rhodocyclus*, compared to representative genomes of close relatives. One hundred rounds of the ‘Rapid bootstrapping’ option of RaxML were used to generate the support values for the phylogenetic tree. The branch length tree scale is defined as the mean number of substitutions per site, which is an average across both nucleotide and amino acid changes. The *Rhodocyclus* genomes are colored differently based on their ANI values (with a species cutoff of 95%). *Allochromatium vinosum* DSM 180^T^ was added as an outgroup.

The ANI data imply that all of the studied strains are indeed members of the genus *Rhodocyclus*, and *Rhodocyclus purpureus* and *Rhodocylus tenuis* are distinct species of this genus. However, the strains of group 2 (DSM 110, DSM 111, and DSM 112) belong to a new species of the genus *Rhodocyclus*. A whole-genome-based phylogenetic tree (Figure 1) supports these findings and shows the group of strains DSM 110, DSM 111, and DSM 112 as very close relatives to each other but apart from the type strains of *Rcy. tenuis* DSM 109^T^ and *Rcy. purpureus* DSM 168^T^. This indicates that this group indeed forms a separate species of the *Rhodocyclus* genus.

**Figure 2:**
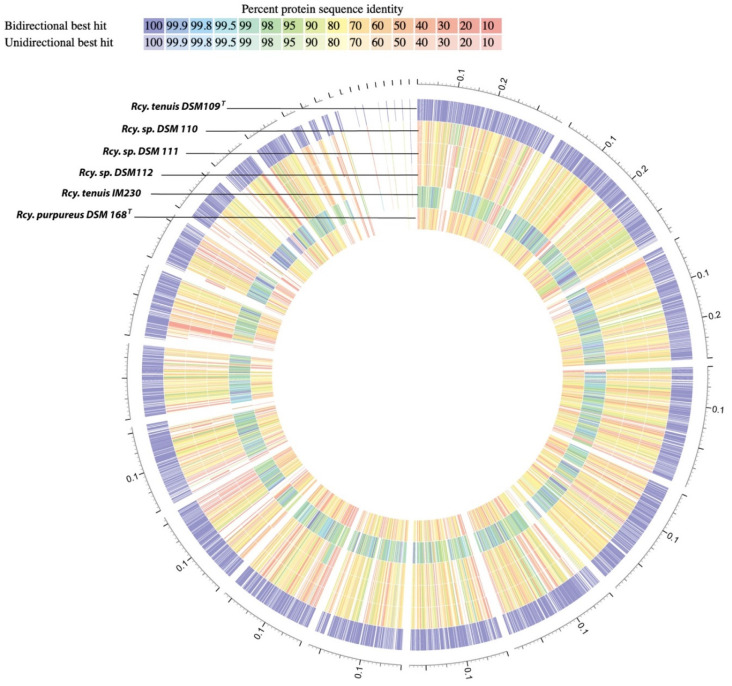
Proteome comparison of *Rhodocyclus* species and relatives based on protein-sequence-based genome comparison using bidirectional BLASTP. *Rcy. tenuis* DSM 109^T^ was used as the reference proteome. The percent protein identity is color-coded for each proteome as compared to the reference proteome. *Rcy. tenuis* IM 230 showed average amino acid identities (AAI) of 97.6% (blue-green), while the *Rcy.* DSM 110, *Rcy*. DSM 111, *Rcy.* DSM 112, and *Rcy. purpureus* proteomes are equidistant from the reference proteome (77–78% identity; yellow-orange).

A protein-sequence-based genome comparison, with the type strain of *Rcy. tenuis* DSM 109^T^ as a reference, provided a complete proteome comparison (Figure 2). This provided amino acid sequence identity for both bi- and unidirectional hits between each genome and the reference genome (color coded in Figure 2). The average amino acid sequence identity (AAI), using only reciprocal (bi-directional) hits, was calculated for each genome, which showed a 78.4% identity with DSM 110 (from 2203 proteins), 77.8% with DSM 111 (from 2184 proteins), and 78.0% with DSM 112 (from 2185 proteins). Similar values were obtained for *Rcy. purpureus* DSM 168^T^ with 77.0% identity (from 1938 proteins). However, a much higher value of 97.6% was found when comparing the *Rcy. tenuis* IM 230 proteome (from 3058 proteins) with the DSM 109 reference proteome. These data correspond well with the other analyses and further support the distinction of the strains DSM 110, DSM 111, and DSM 112 at the species level.

Digital DNA–DNA hybridization analyses (dDDH) showed only a distant relationship between the type strain of *Rcy. tenuis* DSM 109^T^ and the strains DSM 110 (25.0%), DSM 111 (24.9%), and DSM 112 (25.1%). These values are similar to what was obtained between *Rcy. tenuis* DSM 109^T^ and *Rcy. purpureus* DSM 168^T^ (24.8%). The dDDH values between *Rcy. purpureus* and the three strains also places them distantly related, with 22.9% (DSM 110), 23.0% (DSM 111), and 23.0% (DSM 112). On the other hand, the dDDH values amongst the three strains, DSM 110, DSM 111, and DSM 112, showed very high DNA–DNA hybridization values (91–92%). Consistent with the analyses provided above, this places these three strains in a closer relationship with each other than any of the other species, equidistant from *Rcy. tenuis* DSM 109^T^ and *Rcy. purpureus*, DSM 168^T^, supporting the placement of those into a separate species group.

**Figure 3:**
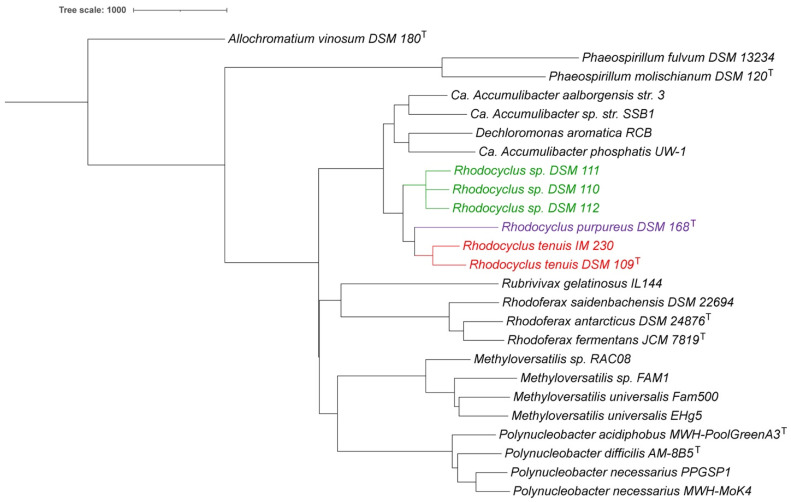
16S rRNA phylogenetic tree for *Rhodocyclus* and related species. The phylogenetic tree was calculated by the neighbor-joining (NJ) method [36] within Jalview [37]. iTOL was used to draw the phylogenetic trees expressed in the Newick phylogenetic tree format [33]. *Allochromatium vinosum* DSM 180^T^ was added as an outgroup. *Rhodocyclus* species were color-coded the same as in Figure 1.

In addition to the whole-genome-based analyses, we also compared the 16S rRNA sequences of all *Rhodocyclus* species and related species. A 16S rRNA-based phylogenetic tree is provided in Figure 3. *Rcy. tenuis* DSM 109^T^ and IM 230 have a 99.4% 16S rRNA identity (1541 nt. overlap), while the three strains DSM 110, DSM 111, and DSM 112 only have 97.4% identity with *Rcy. tenuis* DSM 109^T^ (1541 nt. overlap). They do have 99.9% identity amongst themselves. The *Rcy. purpureus* 16S rRNA is equidistant from *Rcy tenuis* DSM 109^T^ and DSM 110, with 96.6% and 96.0% identity, respectively (1551 nt. overlap). These values are below the proposed species delineation for 16S rRNA comparisons of 98.7% [39] and place *Rcy. purpureus* and the three strains, DSM 110, DSM 111, and DSM 112, on separate clades in the 16S rRNA phylogenetic tree (Figure 3), which is consistent with the whole-genome-based analyses described above.

### 3.2. Cytochrome and High Potential Iron Protein HiPIP Analysis

An interesting aspect of this study is the apparent use of different electron donors to the photosynthetic reaction center. While HiPIP is the normal electron donor to most photosynthetic reaction centers in the *Gammaproteobacteria,* cytochrome *c_2_* fills this role in the *Alphaproteobacteria*. In phototrophic *Betaproteobacteria*, the situation is different. While HiPIP is the usual electron donor in *Rvi. gelatinosus*, a high potential cytochrome *c_8_*, which is induced under aerobic growth, may also participate under some conditions [40]. HiPIP is the electron donor to reaction centers in *Rfx. fermentans* as well [41]. *Rcy. tenuis* is known to utilize both HiPIP and cytochrome *c_8_* in cyclic electron transfer depending on the growth conditions [42].

**Figure 4:**
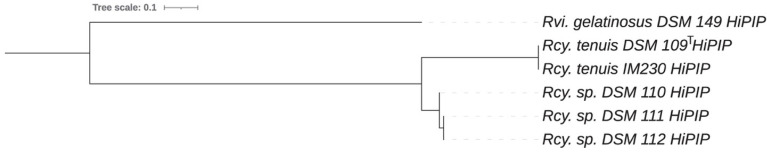
Phylogenetic tree of the HiPIP protein sequences obtained from the *Rhodocyclus* genomes. *Rubrivivax gelatinosus* DSM 149 HiPIP was used as an outgroup.

Soluble electron transfer proteins were previously characterized from strains DSM 109^T^ and DSM 111 and found to be similar to one another but distinct from those of *Rcy. purpureus* [43]. These were described as cytochrome *c_4_* (minor component), cytochrome *c_8_*, cytochrome c-552 (NirB), cytochrome c’, and HiPIP. The latter appears to be absent in *Rcy. purpureus*. A multiple sequence alignment and phylogenetic tree of the HiPIP protein from all *Rhodocyclus tenuis* species (Figure 4) resulted in a phylogenetic relationship that is consistent with the whole genome and ANI comparisons described above. The HiPIP protein sequences from DSM 110, DSM 111, and DSM 112 clearly form a clade on the tree separate from the two sequences of *Rcy. tenuis* DSM 109^T^ and IM230. *Rcy. tenuis* DSM 109^T^ and *Rcy. gracilis* apparently utilize HiPIP as electron donor to the photosynthetic reaction center, but we have now shown that the HiPIP gene, as well as the soluble protein, is lacking in *Rcy. purpureus*, confirming the previous analysis and also suggesting that a cytochrome, presumably *c_8_*, fills the role of mediator between the cytochrome *bc_1_* complex and the PufLMC reaction center.

### 3.3. Nitrogen Metabolism

All species of *Rhodocyclus* apparently produce large amounts of the denitrifying diheme cytochrome NirB [43], which has been shown by the sequence of the protein from *Rcy. tenuis* DSM 109^T^ and DSM 111 [44] and by the fact that all of the *Rhodocyclus* genome sequences in the current study contain the *nir*B gene. However, none of these species apparently have the corresponding denitrifying genes for nitrite, nitric, or nitrous oxide reductases, and NirB apparently assumes a different role. NirB was originally discovered in *Pseudomonas stutzeri* as part of the denitrification pathway, in which it forms a polycistronic mRNA along with the nitrite reductase, NirS (cytochrome cd_1_), and the membrane-bound tetraheme cytochrome, NirT [45]. The implication is that NirT donates electrons to NirB, which in turn reacts with NirS to reduce nitrite to nitric oxide. The normal electron donor to NirS in other pseudomonads is NirM, or C8 as it is also called, and it may be involved in *Ps. stutzeri* as well with NirB enhancing the interaction. Perhaps the role of NirB in *Rhodocyclus* species is to facilitate the interaction of C8 with the cytochrome *bc_1_* complex and PufLMC. This deserves further study.

Consistent with the earlier observations that *Rcy. purpureus* is incapable of fixing molecular nitrogen, while *Rcy. tenuis* DSM 109^T^ showed nitrogenase activity [46], we identified a total of 16 nitrogenase-related PGFams that are absent in *Rcy. purpureus* but present in all of the other *Rhodocyclus* genomes. These include the (Fe-Fe) nitrogenase (alpha, beta, and delta chains), (Mo-Fe) nitrogenase (alpha and beta chains), nitrogen reductase and maturation proteins, two 4Fe-4S nitrogenase-associated ferredoxins, a nitrogenase transcriptional regulator, and several Fe-Mo cofactor assembly proteins.

### 3.4. Cobalamin Metabolism

**Figure 5:**
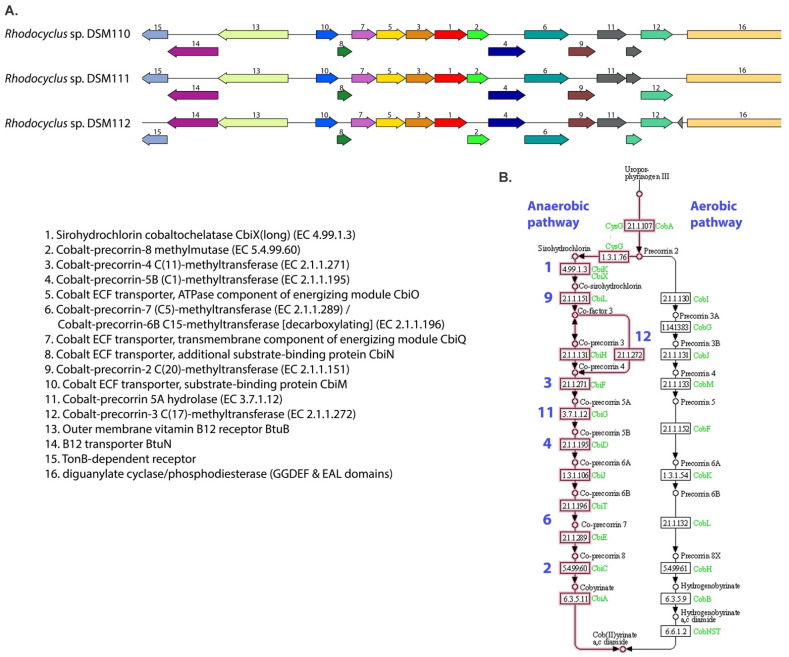
(**A**). Overview of the anaerobic cobalamin genetic pathway that was found in *Rhodocyclus* strains DSM 110, DSM 111, and DSM 112. Synteny plots were generated in PATRIC [22] and genes are colored based on enzymatic family. (**B**). Overview of the anaerobic and aerobic cobalamin metabolic pathways. Enzyme numbering is the same as corresponding gene numbers in (**A**).

When comparing the different genomes, it was found that the 3 *Rhodocylus* strains DSM 110, DSM 111, and DSM 112 all contain at least 12 unique genes related to anaerobic cobalamin (vitamin B_12_) synthesis, which are all missing from the other 3 genomes. These genes code for cobalt–corrin metabolic enzymes and cobalt transporter subunits and are organized in a large gene cluster (Figure 5). The gene synteny of the cluster is conserved in all three strains. It has been known for decades that two pathways exist in nature for the de novo biosynthesis of vitamin B_12_. The pathways differ in the first parts, which involves the corrin synthesis, in which one pathway is anaerobic (as found in *Salmonella typhimurium* and *Bacillus megaterium*) and the other is oxygen-dependent [47,48,49,50]. Figure 5 includes the KEGG pathways and shows that all the gene products necessary for anaerobic corrin synthesis (cobalt-containing modified tetrapyrrole component of vit. B_12_) are present in the *Rhodocyclus* gene cluster. The ATP-dependent transport system encoded by the corrin biosynthetic operon in *S. typhimurium* (CbiMNQO), mediates transport of cobalt ions for the B_12_ synthesis [47]. In addition, vitamin B_12_ and other corrinoids are actively transported using the TonB-dependent outer membrane receptor BtuB in complex with the ABC transport system BtuFCD [51]. The *Rhodocyclus* gene cluster also contains a unique outer membrane vitamin B_12_ receptor BtuB and transporter BtuN (Figure 5).

In addition to the PATRIC PGFam comparison, we also checked for the presence of each of these cobalamin metabolic genes individually with BLAST and only found a truncated version of one of the enzymes (gene 6 in Figure 5) in the *Rcy. purpureus* and *Rcy. tenuis* DSM 109^T^ and IM230 genomes. This could be an indication that the anaerobic cobalamin biosynthetic pathway was lost during evolution, although further analysis that includes more evolutionary divergent species would be needed to confirm this.

The lack of this anaerobic cobalamin metabolism pathway explains why Pfennig originally described *Rcy. purpureus* as a vitamin B_12_-requiring member of the *Rhodospirillaceae* [9]. According to this genome comparison, strains DSM 109^T^ and IM230 would also need cobalamin as a growth factor as they grow anaerobically; however, the need for this has not been described. Either way, the presence of this pathway exclusively in the three *Rcy.* strains DSM 110, DSM 111, and DSM 112 further distinguishes them genetically from the other *Rhodocyclus* species.

### 3.5. Chemotaxis and Motility

*Rcy. purpureus* was found to be non-motile [9], while *Rcy. tenuis* was highly motile [10]. We found 31 flagella related PGFams that were absent from the *Rcy. purpureus* genome but present in all the other *Rhodocyclus* strains. These included all of the biosynthetic, structural and regulatory proteins for flagella assembly. In addition, there are at least 12 unique chemotaxis related PGFams present, including, 2 CheWs, CheA, CheB, CheR, CheZ, CheY, CheD, CheV and MCPs. This chemotaxis gene cluster is directly upstream of the flagella genes in all of these genomes. The presence of these genes confirms that all of the *Rhodocyclus* species, expect *Rcy. purpureus*, are motile, and are expected to perform chemotaxis.

### 3.6. RuBisCo

Genomes of *Rcy. tenuis* and the new species *Rhodocyclus gracilis* have two forms of RuBisCo. One gene encodes a single 463 aa. protein (PGFam_00048972) and is included in a gene cluster with *cbb*Y-*rbc*-*cbb*R-fructose-1,6-bisphosphatase- phosphoribulokinase. This cluster is also present in *Rcy. purpureus*. The second system of RuBisCo consists of a large (474 aa.; PGFam_00048973) and small subunit (118 aa.; PGFam_00048975), included in a gene cluster of *cbb*R-*rbc*L-*rbc*S-*cbb*Q-*cbb*O, which is absent from *Rcy. purpureus* DSM 168^T^. The presence of multiple RuBisCo forms is not uncommon, and the sequences and structural gene organization indicate that the *Rhodocyclus* single rubisco gene belongs to Form II, while the rbcL and rbcS resemble Form I RuBisCo found in other proteobacteria [52].

**Figure 6:**
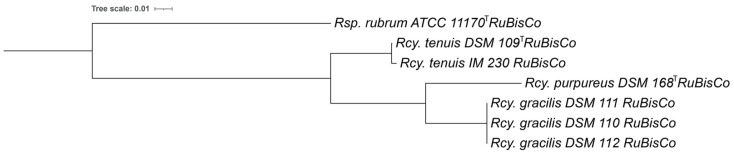
Phylogenetic tree based on multiple sequence alignment of the RuBisCo protein sequences, obtained from the *Rhodocyclus* genomes (PGFam_00048972). *Rhodospirillum rubrum* ATCC 11170^T^ RuBisCo was added as an outgroup.

The translated protein sequence of the single RuBisCo gene (PGFam_00048972) that is present in all *Rhodocyclus* genomes, was used for a phylogenetic comparison (Figure 6). The RuBisCo protein sequence of *Rcy. gracilis* strains is closer related to *Rcy. purpureus* DSM 168^T^, with 94.1% identity (98.3% similarity) than to the *Rcy. tenuis* DSM 109^T^ with 91.6% identity (97.8% similarity). As expected, the *Rcy. tenuis* IM 230 RuBisCo shows a high protein identity to the one from *Rcy. tenuis* DSM 109^T^ (99.8% identity, 100% similarity), while the *Rcy. tenuis* DSM 109^T^ and *Rcy. purpureus* sequences were less similar (90.8% identity, 96.3% similarity). The phylogenetic tree based on the RuBisCo multiple protein sequence alignment (Figure 6) shows a similar topology as the whole genome, 16SrRNA and cytochrome-based comparisons, and further supports the proposed species differentiation.

### 3.7. Taxonomic Considerations

A limited number of strains of *Rhodocyclus* isolates have been studied and compared in several studies during the past decades. Strains were isolated from various freshwater lakes in Germany by Hanno Biebl and Norbert Pfennig and their properties compared [12]. Although two color variants were recognized, a brownish-red colored one, including the type strain of *Rcy. tenuis* DSM 109^T^, and a red-colored or purple-violet one, including strains now assigned to *Rcy. gracilis*, no clear differentiation of groups of strains was made [12].

The different colored cultures showed differences in absorption spectra in the carotenoid region (450–550 nm) [12] and also revealed a different carotenoid composition [13]. The type strain of *Rcy. tenuis* DSM 109^T^ has carotenoids of the spirilloxanthin series with major portions of lycopene, rhodopin and anhydro-rhodovibrin and spirilloxanthin as the final product of this pathway. The purple-violet-colored DSM 110, DSM 111, and DSM 112 contain significant amounts of rhodopinal, rhodopinol, and lycopenal in addition to rhodopin and lycopene but lack spirilloxanthin, rhodovibrin, and anhydro-rhodovibrin [13], a property they share with *Rcy. purpureus* DSM 168^T^ [13].

Studies on the lipopolysaccharides of eight isolates assigned to *Rhodocyclus tenuis* revealed a common pattern of sugars characterized by the presence of glycerol-mannoheptose, glucose, arabinose, 2-keto-3deoxyoctonoate and glucosamine in all studied strains [14]. While the presence of D-galactosamine was found only in those strains assigned now to *Rcy. gracilis* (DSM 110, DSM 111, DSM 112), the type strain of *Rcy. tenuis* DSM 109^T^ (2761) lacked D-galactosamine and in turn had quinovosamine as a strain-specific sugar [14].

Based on the unique genomic and genetic features of the *Rhodocyclus* strains described above, it is clear that strains DSM 110, DSM 111, and DSM 112 belong into a single species which is separate from *Rcy. tenuis* and *Rcy. purpureus*. Of the strains compared in the present study, only strains DSM 109^T^ and IM 203 remain as strains of *Rcy. tenuis*. For the strains recognized as a new species, the name *Rhodocyclus gracilis* sp. nov. is proposed.

The characteristic properties that distinguish strains of *Rcy. gracilis* from *Rcy. tenuis* are the utilization of ethanol [12] and the presence of carotenoids of the rhodopinal series [13], which coincides with the purple-violet color of *Rcy. gracilis*. The pH optimum of *Rcy. gracilis* is slightly lower, at pH 6.1–6.4, compared to the pH 6.7 of *Rcy. tenuis* [12]. The genomes are different in size, 2.93–2.98 Mb for *Rcy. gracilis* and 3.65–3.85 Mb for *Rcy. tenuis* (Table 1), and the G + C content is 64.5 mol% in *Rcy. gracilis* and 64.7 mol% in *Rcy. tenuis* (Table 1).

Due to the lack of a clear differentiation of groups of strains of *Rcy. tenuis* in previous studies, the properties of both *Rcy. gracilis* and *Rcy. tenuis* have been listed as the properties of *Rcy. tenuis* in the literature [12,13,14,53]. Consequently, the species description of *Rcy. tenuis* should be emended accordingly.

Characteristic for all *Rhodocyclus* species is the presence of phosphatidyl glycerol (PG), phosphatidyl ethanolamine (PE), diphosphatidyl glycerol (CL), and an ornithine lipid as major polar lipids; the dominance of C-16 fatty acids (33–36% C-16:0 and 43–50% C-16:1) and minor amounts of the C-18 fatty acids (<0.5% C-18:0 and 14–18% C-18:1); as well as ubiquinone Q-8 and menaquinone MK-8 as major quinone components [16].

#### 3.7.1. Description of *Rhodocyclus gracilis* sp. nov

##### *Rhodocyclus gracilis*. gra’ci.lis M.L. neut. adj. *gracilis* Slender

Cells are weakly curved, 0.3–0.5 μm wide and 1.5–5 μm long, motile by polar flagella and divide by binary fission. Cultures grown anaerobically in the dark are red to purple-violet in color and have absorption maxima at 377–378, 469, 495–500, 529–533, 590–592, 798–801, and 856–858 nm. Photosynthetic pigments are bacteriochlorophyll-a and carotenoids of the rhodopinal series. Internal photosynthetic membranes are present as small finger-like intrusions of the cytoplasmic membrane.

Growth occurs preferably under phototrophic conditions anaerobically in the light. Under these conditions, organic carbon compounds are used as carbon and energy sources. The sources utilized are acetate, propionate, butyrate, valerate, caproate, lactate, pyruvate, fumarate, malate, succinate, and ethanol. Some strains may use pelargonate and yeast extract. Not utilized are tartrate, citrate, benzoate, methanol, glycerol, glucose, fructose, mannitol, alanine, glutamate, aspartate, arginine, thiosulfate, and sulfide. Sulfide is growth inhibitory at 2 mM. Chemotrophic growth under aerobic dark conditions is possible. Aerobically grown cells are colorless, and the aerobic Mg-protoporphyrin IX monomethyl ester oxidative cyclase is absent. Photolithotrophic growth with hydrogen as an electron source may be possible. Ammonium chloride and dinitrogen are used as nitrogen sources. Growth factors may be required. Mesophilic freshwater bacterium with optimum growth at 30 °C and pH 6.1–6.4 (pH range 4.9–8.2). Habitats are freshwater lakes and peat bogs. The habitat of the type strain DSM 110 is a dystrophic pond in the Black Forest (Germany).

The type strain has a G + C content of the DNA of 64.5 mol% (genome analysis) and a genome size of 2.93 Mb. The type strain is deposited with the Deutsche Sammlung von Mikroorganismen und Zellkulturen as DSM 110^T^ (Pfennig 3760) and the Japan Collection of Microorganisms Riken BRC.

Gene bank accession number of the 16S rDNA sequence of the type strain OM179767 and of the genome WIXJ00000000.

#### 3.7.2. Emended Description of *Rhodocyclus tenuis* Imhoff, Trüper and Pfennig 1984, 341.^VP^ (*Rhodospirillum tenue* Pfennig 1969, 619.^AL^)

##### te’nu.is. L. masc. adj. *tenuis* Slender, Thin

Cells are weakly curved spirals, highly motile by polar flagella. They are 0.3–0.5 µm wide and 1.5–6.0 µm long, sometimes even longer. One complete turn of a spiral is about 0.8–1.0 µm wide and 3 µm long. Photosynthetically grown cells are brownish-red and have absorption maxima at 378–380, 465, 492–495, 528, 592–594, 799–801, and 868–871 nm. Photosynthetic pigments are bacteriochlorophyll-a esterified with phytol and carotenoids of the spirilloxanthin series.

Growth occurs preferably under anoxic conditions in the light with organic carbon compounds as carbon and electron sources. Photolithotrophic growth with molecular hydrogen is possible. Chemotrophic growth is possible under microoxic to oxic conditions in the dark. Aerobically grown cells are colorless or pale red. Under phototrophic growth conditions organic carbon compounds are used as carbon and energy sources. The sources utilized are acetate, butyrate, valerate, caproate, lactate, pyruvate, fumarate, malate, and succinate. Pelargonate and propionate may be used by some strains. Not utilized are formate, ethanol, tartrate, citrate, benzoate, cyclohexane carboxylate, methanol, glycerol, glucose, fructose, mannitol, alanine, glutamate, aspartate, arginine, thiosulfate, and sulfide. Sulfide is growth-inhibitory at 2 mM. The nitrogen sources utilized are aspartate, glutamate, glutamine, ammonia, and dinitrogen and also casamino acids, peptone, yeast extract, alanine, arginine, lysine, methionine, serine, threonine, and urea. Sulfate, glutathione, cysteine, thiosulfate, and also sulfite and sulfide at low concentrations can serve as assimilatory sulfur sources. Growth factors are not required. Growth is stimulated, however, in the presence of complex organic nutrients or yeast extract and some strains may need vitamin B_12_.

Mesophilic freshwater bacterium with optimum growth at 30 °C and pH 6.7–7.4.

Habitat: freshwater ponds, sewage ditches.

The type strain has a G + C of the DNA of 64.7 mol% (genome analysis) and a genome size of 3.85 Mb.

Type strain: ATCC 25093, DSM 109 (Pfennig: 2761, Grünenplan).

Gene bank accession number of the 16S rDNA sequence of the type strain: D16208.

Gene bank accession number of the genome sequence of the type strain: SSSP00000000.

## Figures and Tables

**Table 1:** Overview of genome features of all of the *Rhodocyclus* genome sequences.

Species	Genome Size	GC Content	Contigs	Coverage	CDS	tRNAs	Reference	Genbank Accession #
								
***Rhodocyclus tenuis DSM109***	3.85 Mb	64.7	30	100x	3605	51	*Wang et.al., 2020*	SSSP00000000
***Rhodocyclus tenuis IM230***	3.65 Mb	64.7	28	93x	3359	51	*this study*	NRRZ00000000
								
***Rhodocyclus* sp. *DSM110***	2.93 Mb	64.5	27	54x	2735	47	*this study*	WIXJ00000000
***Rhodocyclus* sp. *DSM111***	2.93 Mb	64.4	30	55x	2705	47	*this study*	WJED00000000
***Rhodocyclus* sp. *DSM112***	2.98 Mb	64.5	27	73x	2768	47	*this study*	JAATWB000000000
								
***Rhodocyclus purpureus DSM168***	3.62 Mb	66.1	69	81x	3600	51	*this study*	NHRX00000000

**Table 2:** Whole-genome-based average nucleotide identity (ANI) of *Rhodocyclus* species and relatives. ANI values above the species cutoff of 95% are shown in bold.

*Rhodocyclus tenuis* DSM 109^T^								
**97.1**	*Rhodocyclus tenuis* IM 230							
80.3	80.3	*Rhodocyclus purpureus* DSM 168^T^						
79.7	79.4	79.9	*Rhodocyclus* sp. DSM 111					
79.8	79.4	77.8	**98.8**	*Rhodocyclus* sp. DSM 110				
79.7	79.5	77.9	**98.9**	**98.9**	*Rhodocyclus* sp. DSM 112			
73.3	72.9	73.1	72.5	72.5	72.5	*Ca. Accumulibacter phosphatis* UW1		

## Data Availability

This Whole Genome Shotgun project has been deposited at DDBJ/ENA/GenBank under the accession numbers provided in Table 1.
